# HIGH-er Order Cognitive Functioning: Metacognition and Cannabis Use in Older Adults

**DOI:** 10.1093/geroni/igaf122.2562

**Published:** 2025-12-31

**Authors:** Elizabeth Anquillare, Caleb Moyer, Juliamaria Coromac-Medrano, Rachel Thayer

**Affiliations:** University of Colorado Colorado Springs, Colorado Springs, Colorado, United States; University of Colorado Colorado Springs, Colorado Springs, Colorado, United States; University of Colorado Colorado Springs, Colorado Springs, Colorado, United States; University of Colorado Colorado Springs, Colorado Springs, Colorado, United States

## Abstract

Previous research shows metacognition remains stable in older adulthood, despite age-related declines in other cognitive domains. Although cannabis has been demonstrated to have deleterious effects on executive function, which overlaps with metacognition in both construct and underlying neural mechanisms, metacognition has not yet been studied in the context of older adult cannabis use. Additionally, metacognition refers to several skills including self-monitoring and strategy implementation which may have unique relationships with cannabis use. This study aimed to explore associations among subjective cognitive functioning, strategy use, and objective cognitive functioning in a pilot sample of older adults using cannabis (OAUC). Participants were OAUC (N = 17, Mage=68.29, SD = 5.61, range 60-79 years), of whom 59% identified as women and 94% identified as White, with an average 15.94 years of education (SD = 2.38). Participants completed online subjective cognition surveys and a virtual neuropsychological assessment battery. Subjective cognitive functioning and strategy use were not significantly associated, r=.11, ns. Greater strategy use was associated with lower objective processing speed and executive function scores (-.60≤r≤-.40). Better subjective cognitive functioning was associated with worse verbal learning and self-monitoring scores (-.41≤r≤-.31). OAUC may require greater strategy use in the context of worse objective functioning possibly related to cannabis use. Additionally, OAUC may have inflated confidence in their cognitive ability due to successful strategy use, resulting in discrepancies between subjective and objective cognition. Considering the importance of self-monitoring and strategy use in daily functioning (e.g., medication management), more research is needed to understand the role of cannabis in metacognitive ability in older adults.

